# Mid-term functional outcome after the internal fixation of distal radius fractures

**DOI:** 10.1186/1749-799X-7-4

**Published:** 2012-01-26

**Authors:** Joideep Phadnis, Alex Trompeter, Kieran Gallagher, Lucy Bradshaw, David S Elliott, Kevin J Newman

**Affiliations:** 1 Orthopaedic Department, Frimley Park Hospital, Camberley, GU16 7UJ, UK; 2 Orthopaedic Department, Kingston Hospital, Galsworthy Road, KT2 7QB, UK; 3 Orthopaedic Department, St Peters Hospital, Chertsey, KT16 0PZ, UK; 4 Medical statistics department, Cambridge University, CB2 1TN, UK

**Keywords:** Distal Radius, Fracture, Internal fixation: functional outcome

## Abstract

**Background:**

Distal radius fracture is a common injury with a variety of operative and non-operative management options. There remains debate as to the optimal treatment for a given patient and fracture. Despite the popularity of volar locking plate fixation, there are few large cohort or long term follow up studies to justify this modality. Our aim was to report the functional outcome of a large number of patients at a significant follow up time after fixation of their distal radius with a volar locking plate.

**Methods:**

180 patients with 183 fractures and a mean age of 62.4 years were followed up retrospectively at a mean of 30 months (Standard deviation = 10.4). Functional assessment was performed using the Disabilities of the Arm, Shoulder and Hand (DASH) and modified MAYO wrist scores. Statistical analysis was performed to identify possible variables affecting outcome and radiographs were assessed to determine time to fracture union.

**Results:**

The median DASH score was 2.3 and median MAYO score was 90 for the whole group. Overall, 133 patients (74%) had a good or excellent DASH and MAYO score. Statistical analysis showed that no specific variable including gender, age, fracture type, post-operative immobilisation or surgeon grade significantly affected outcome. Complications occurred in 27 patients (15%) and in 11 patients were major (6%).

**Conclusion:**

This single centre large population series demonstrates good to excellent results in the majority of patients after volar locking plate fixation of the distal radius, with complication rates comparable to other non-operative and operative treatment modalities. On this basis we recommend this mode of fixation for distal radius fractures requiting operative intervention.

## Background

Fractures of the distal radius are common [1-3]. Increasing incidence of these injuries may be attributed to an ageing population (osteoporotic fractures) and the growing participation in outdoor pursuits (higher energy fractures) [4,5]. Various operative and non-operative modalities are available for treatment, but there remains controversy as to the optimal method for a given patient and fracture type [2,3]. Volar locking plate fixation has become increasingly popular, especially in patients with osteoporotic bone and this is reflected by the number of implants available on the market [6]. Despite this there are few long term, comparative or large cohort studies looking specifically at the functional outcome after volar locking plate fixation.

The aim of this study was to review a large group of patients treated with volar locking plate fixation of their distal radius fracture and to assess their functional outcome at a significant follow up time. Secondary aims were to establish if any individual factors affected this outcome and compare our results with those of other studies.

Our hypothesis was that functional outcome and complication rate after internal fixation of the distal radius with a volar locking plate would be comparable to, or better than other reported treatment modalities.

## Methods

We conducted a retrospective study of patients with fractures of the distal radius managed by internal fixation using the Stryker Matrix Volar Locking Plate (Stryker Leibinger GmbH & Co. Germany) between June 2004 and October 2007 treated in a single centre. Indications for surgery were displaced intra-articular fractures with post reduction articular step of > 2 mm; radial shortening of > 3 mm or > 15 degrees of saggital plane angulation (as measured from the anatomical volar tilted position)[2]. Additionally, fractures with features indicative of instability or poor outcome such as metaphyseal comminution and unsatisfactory radio-carpal alignment were treated surgically [7]. Finally, patients who originally underwent non-operative treatment in plaster but whose fracture displayed one of the above stated parameters for surgery at follow up, and underwent subsequent volar plate fixation were included. Exclusion criteria were fractures treated with alternative instrumentation, fractures over 4 weeks old at the time of surgery, and non-trauma operations such as corrective osteotomies.

### Patients

Using the departmental trauma database, 221 suitable patients were identified for inclusion in the study. One patient was unwilling to participate; six were deceased; 26 were not contactable and eight were unable to participate for medical reasons. The remaining 180 patients with a total of 183 fractures were followed up by means of case note and operative record analysis, radiographic assessment and functional questionnaire.

### Outcome assessment

Case notes were used to establish all demographic details including; mechanism of injury; time to surgery; post-operative immobilisation; surgeon grade and intra-operative details. Complications were also recorded and confirmed with the patients.

The functional questionnaire was telephone based, and utilised the 'quick' Disabilities of Arm, Shoulder and Hand (Quick DASH) score [8-11], and the modified MAYO wrist score [12]. The Quick DASH has been shown to be interchangeable with the traditional DASH score and is simpler to perform [8,10,11]. Both MAYO and DASH are scored from 0-100 but in the DASH score, a score of 100 constitutes the worst outcome whereas in the MAYO score this is equivalent to the best outcome. There are no defined parameters of what constitutes an excellent, good, satisfactory or poor score in the DASH system merely that a score of zero indicates no disability and a score of 100 indicates complete disability. We hence set these parameters ourselves for descriptive purposes. Such parameters are pre-defined in the MAYO score (table 1). JP and KG who were blinded to the patient's demographic details performed the functional assessment for all patients.

**Table 1:** Outcome grading for Quick DASH and MAYO wrist scores

	DASH	MAYO
**EXCELLENT**	0-5	90-100

**GOOD**	6-15	80-90

**SATISFACTORY**	15-35	60-80

**POOR**	> 35	< 60

Pre-operative radiographs were classified according to the AO-ASIF classification system [13] and post- operatively were assessed for fracture union. Fracture union was determined by the presence of bridging trabeculae on the AP and Lateral view radiographs or by complete obliteration of the fracture line [14]. These findings were correlated with the case notes to determine whether there was pain on physiologic loading or on palpation before fracture union was declared.

### Statistical methods

Categorical variables and baseline demographic data are described using frequencies and percentages. Continuous variables with a symmetric distribution are presented using means and standard deviations (SD) and continuous variables with a skewed distribution are presented using the median and inter-quartile range (IQR). Demographic variables such as age and gender and operation related variables such as surgeon grade, fracture type and time to surgery were considered to establish if any of these factors affected functional outcome. Spearman's rank correlation coefficient was used to assess the association between continuous variables and non-parametric tests (Mann-Whitney and Kruskal-Wallis) were used to test for differences in outcome between groups. All tests were two-sided with the level of significance set at 0.01 to take account of multiple testing.

### Surgical technique

Surgery was performed under general or regional anaesthesia with use of an arm tourniquet and administration of antibiotics according to local policy. A standard volar approach through the bed of Flexor Carpi Radialis was performed. The Stryker Matrix Smartlock volar locking plate (Stryker Leibinger GmbH & Co. Germany) was used in all cases. This is a low profile, titanium plate incorporating 20 degree variable angle locking for all screws. Skin closure and postoperative immobilisation was according to the operating surgeon's preference. Bone grafting and carpal tunnel decompression were not routinely performed, although carpal tunnel decompression was done at the time of internal fixation in 10 patients at the discretion of the operating surgeon. Reasons for decompression included pre-existing carpal tunnel syndrome (n = 2); pre-operative symptoms of median nerve compression (n = 5) and excessive intra-operative swelling (n = 3). Ulna styloid fractures were not treated operatively in any patients. Distal Radio-Ulnar Joint (DRUJ) instability was documented in only one case and was treated with Kirschner wire stabilisation for six weeks.

## Results & discussion

**Figure 1:**
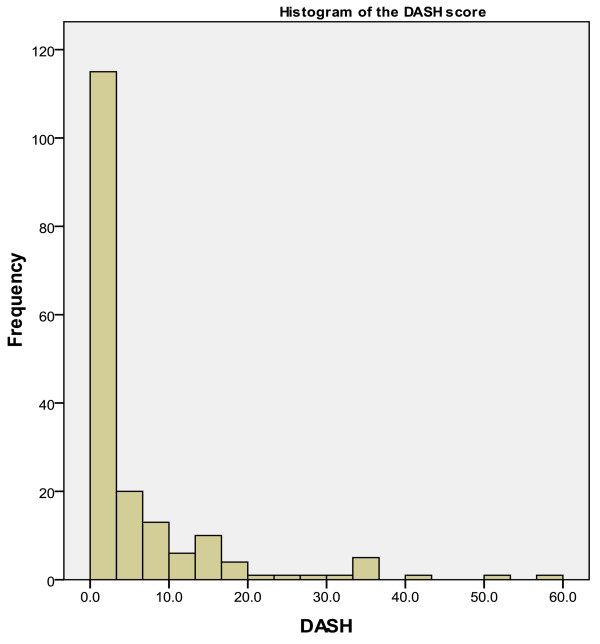
**Histogram showing distribution and frequency of DASH scores**.

**Figure 2:**
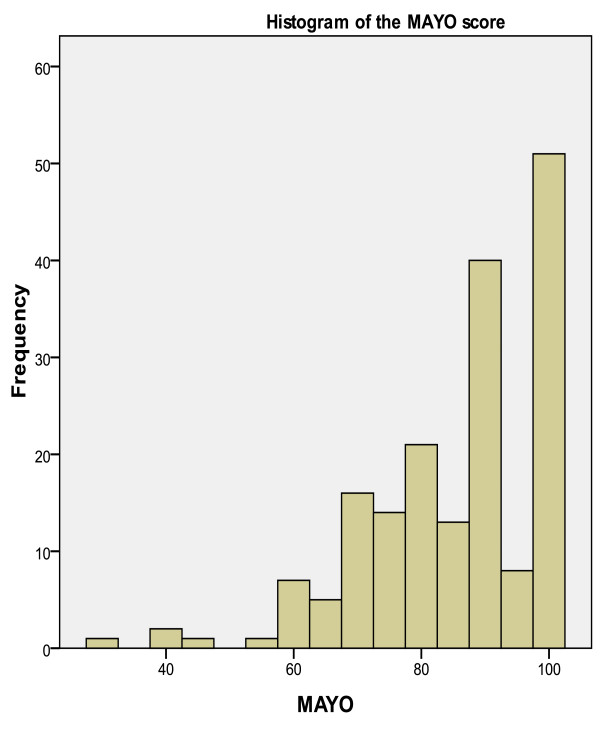
**Histogram showing distribution and frequency of MAYO scores**.

The median DASH score for all patients was 2.3 (IQR 0-6.4) and the median MAYO score was 90 (IQR 75-100). The MAYO and DASH scores were good or excellent in 133 patients (74%), satisfactory in 41 (23%) and poor in six patients (3%). There was a strong relationship between the two scoring systems with virtually no variation in outcomes. Figure 1 and 2 show the distribution and frequency of DASH and MAYO scores for the whole group.

### Possible variables affecting functional outcome

132 patients were female (73%) and 51 were male (27%). There was little variation in the MAYO score between men and women, however the DASH score for women was more variable than in men. A Mann-Whitney test showed some indication of a higher DASH score in women than in men (p = 0.025), however when taking into account multiple testing using the Bonferroni adjustment, this is was not considered statistically significant.

Mean age was 62.4 years (range16-93; SD 17.9). No relationship was seen between age and either the MAYO or DASH score (Spearman's correlation coefficient, MAYO 0.03; DASH 0.04).

Mean time to surgery was 8 days (range 0-28 days; SD 6.4). No relationship was seen between time to surgery and either the MAYO or DASH score (Spearman's correlation coefficient, MAYO -0.09; DASH 0.1).

Mean time at follow up was 30 months post operatively (13-53 months; SD 10.4). In total, 62 patients (34%) were followed up in their second postoperative year; 69 (38%) in year three; 42 (23%) in year four and seven (4%) in year five. Patients did not have significantly different functional outcome according to their year of follow up.

The primary surgeon was a consultant in 56 cases (31%) and was a middle grade (trainee registrar or staff grade surgeon) in 127 cases (69%). In total 26 different surgeons performed all operations. Using the Kruskal-Wallis test there was no evidence of a difference in MAYO (p = 0.38) or DASH score (p = 0.73) for the different surgeon grades.

Post-operative immobilisation for between four and six weeks was instituted in 76 patients (42%) as opposed to immediate graduated mobilisation in the remaining 107 patients (68%). This was surgeon dependent and there was no significant discrepancy between the two groups in terms of fracture type or patient age. There was no statistical difference between the groups in terms of functional outcome (DASH p = 0.06; MAYO p = 0.11) or in the incidence Chronic Regional Pain Syndrome (CRPS) (p = 0.09).

Table 2, shows a breakdown of the differing fracture types. Mean DASH and MAYO scores were comparable for all fracture types and there was no evidence of a difference in MAYO (p = 0.90) and DASH scores (p = 0.80) for the different fracture types (Kruskal-Wallis).

**Table 2:** Classification of fracture types and corresponding DASH and MAYO scores

Fracture Type (AO-ASIF)	Frequency	Percentage	Combined Frequency	Combined Percentage	Combined Mean DASH	Combined Mean MAYO	Combined Median DASH	Combined Median MAYO
23-A1	0	0%	94	51.4%				
						
23-A2	34	18.6%			6.5	84.0	2.3	90
						
23-A3	60	32.8%			SD = 11.3	SD = 9.3	IQR = 0-6.8	IQR = 80-100

23-B1	7	3.8%	18	9.8%				
						
23-B2	1	0.5%			5.4	85.4	3.4	82.5
						
23-B3	10	5.5%			SD = 8.6	SD = 13.3	IQR = 0-5.7	IQR = 70-100

23-C1	12	6.6%	71	38.8%				
						
23-C2	35	19.1%			3.5	88.1	1.7	90
						
23-C3	24	13.1%			SD = 6.5	SD = 9.32	IQR = 0-5	IQR = 75-100

**Total**	**183**	**100**	**183**	**100**				

### Radigraphic analysis

133 patients (74%) had post-operative radiographs available for analysis. This was because there was a change over to electronic imaging in the first year of the study period making it difficult to access all radiograph hard copies.

Overall mean time to fracture union was 8.4 weeks (6-28 weeks). Table 3, shows time to union by fracture type. Although there was a trend towards increasing union time with higher energy fracture type, this did not prove to be significant (Kruskal-Wallis p = 0.35). There were no cases of non-union.

**Table 3:** Time to Fracture Union with varying fracture type

Fracture Type AO-ASIF	Frequency	Percentage	Mean time to union (weeks)	Standard Deviation (weeks)	Median time to union (weeks)	Inter Quartile Range (weeks)
23-A2	15	11.3%	7.4	1.6	7	6-8

23-A3	50	37.6%	7.7	2.5	7	6-8

23-B	7	5.3%	8.29	3.6	7	6-9

23-C1	8	6.0%	6.63	0.7	6.5	6-7

23-C2	30	22.6%	8.73	3.0	8	6-10

23-C3	23	17.3%	10.74	7.4	8	6-17

Total	133	100%				

### Complications

In total, 27 patients (15%) suffered a postoperative complication. These are outlined in table 4. 11 patients (6%) sustained a major complication, defined as deep infection, tendon rupture, acute carpal tunnel syndrome and chronic regional pain syndrome. Two patients developed persistent tingling in the median nerve distribution post operatively. Neither of these had a motor deficit or pre-operative median nerve dysfunction. Both were expediently taken back to theatre within 12 hours of surgery for carpal tunnel decompression, which resolved their symptoms. Of the three tendon ruptures, two were of Extensor Pollicis Longus (EPL) and one was of Flexor Pollicis longus (FPL). One EPL rupture had protruding screws dorsally whereas the other did not. The case with the FPL rupture did not have a prominent plate radially however at the time of removal it was found that the radial screw had not been fully locked into the plate, making its prominence the likely cause for rupture. The mean DASH and MAYO scores for patients suffering a postoperative complication were 9.0 and 80.3 respectively. Plate removal was performed in 17 patients (9%). This was for deep infection in one case; tendon rupture in three cases; prominent dorsal screws in three cases; late carpal tunnel syndrome in two patients and at the patients request in seven cases. There were no further incidences of plate related complications.

**Table 4:** Post-operative complications

Major Complications		Minor Complications	
Unresolved CRPS	5	Resolved CRPS	11

Tendon Rupture	3	Late carpal tunnel syndrome	2

Acute Carpal Tunnel Syndrome	2	Superficial Infection	2

Deep Infection	1	Hypertrophic scar	2

**Total**	**11 (6%)**	**Total**	**16 (9%)**

## Discussion

We present one of the largest single centre case series of this kind, with good or excellent outcome in the majority of patients at 30 months follow up. While there are long term studies looking at the outcomes of patients treated non-operatively [3,7], with external fixation [15], and percutaneous fixation [16.17], there are few studies documenting the functional outcome after volar plate fixation beyond 2 years post operatively. Rozental et al. showed mostly good and excellent functional outcomes in 45 patients at 17 months mean follow up [18]. Similar larger series [19,20] have reviewed the outcome of volar plate fixation in cohorts of 150 (24 months follow up) and 114 (12 months) patients respectively. Like our study these both showed good to excellent functional outcome using the DASH score. Of note however, there was a 22% loss to follow up at 24 months in one series [19].

Our study is limited by the fact that it is retrospective. This did not allow us to follow up patients at several time points after surgery and correlate progression in functional score with time. Other authors have demonstrated that improvement in functional scoring is particularly applicable to the first year after surgery [21,22]. Goldfarb et al. and Catalano et al. followed up the same cohort of patients at a mean of 7 and 15 years after open reduction and stabilisation with wires and showed a long term progressive increase in functional score [23,24]. However, there is no current data to show whether a similar improvement is seen after volar locking plate fixation. The mean DASH scores in our study are better than in the corresponding papers mentioned and we feel this reflects the longer follow up time and infers that functional scores may continue to improve with time.

We used the MAYO wrist score alongside the DASH score to assess functional outcome. There was no discrepancy between the two scoring systems in terms of grading outcome. Although the two scoring systems are not directly comparable, the use of two systems reduces the likelihood of errors generated by the use of a single system. We used the quick DASH score which is comprised of 11 questions and has been shown to have similar cross-sectional and test-retest reliability to the traditional 30 point full DASH score [8-11]. Although not significantly different, Gummesson et al. showed quick DASH scores tended to be higher than DASH scores [10]. This reassures us that the scores we recorded are reliable and comparable to other studies.

We had expected fracture type, surgeon grade, time to surgery or patient age to be possible factors, which affected functional outcome, however we were not able to demonstrate this. This is actually consistent with the findings of other studies [21,22]. It is likely that the fact that we assessed patients at a single time point long after their original surgery may have allowed any initial discrepancies to even out. We suspect that if we had followed up patients prospectively at multiple time points, factors such as post-operative immobilisation, age and time to surgery would have been more influential on functional outcome. Nevertheless, it is reassuring that in the longer term these factors do not appear detrimental to functional outcome. With regard to surgeon grade and fracture type, the results may be masked by the fact that more senior surgeons tackled the more complex fractures, thus making it difficult to assess the true influence of surgeon grade.

Only one type volar locking plate was used for all patients in this series as this was the only implant available at our centre during the study period. Subsequent development has lead to the design of many newer plates with a variety of features including different locking mechanisms, material properties and fragment specific designs. The use of newer plates may have changed outcomes in some way, however the basic principles of fracture reduction, stable fixation and respect for the soft tissues remain paramount regardless of implant.

Many studies reviewing various methods of fixation look at radiographic parameters that affect outcome, however few if any have looked at surgeon grade or time to surgery as we have. In a large radiographic study, Mackenney et al. showed that age over 80 years; metaphyseal comminution and positive ulna variance were the main predictors of instability. This and poor radio-carpal alignment were shown to be associated with poor outcome [7]. However this study did not look at results after fixation with a volar locking plate, which has specific design applications for use in osteoporotic unstable fracture patterns. We chose not to assess radiographic parameters such as residual intra-articular step, correction of normal distal radial anatomy and presence of post traumatic osteoarthritis as we were interested purely in patient centered outcome and these radiographic features have consistently been shown not to correlate with functional outcome [4,21-24].

At the time of the study and currently, it is not our practice to treat Ulna styloid fractures surgically. This is based on the fact that they have not conclusively been shown to alter functional outcome [2,25,26].

The only factor that was associated with a poorer functional outcome was the development of a post-operative complication. The 27 patients with a post-operative complication had both DASH and MAYO scores considerably worse than the other patients, and those with what we classed as a major complication (10 patients) had a much worse functional score. The two patients who acutely developed post operative carpal tunnel syndrome were both females under the age of 60 years. Neither had preoperative symptoms. One had a 23-C2 type fracture whilst the other had a 23-A2 fracture. Ring et al. suggested that younger women with significantly translated fractures may be at higher risk of carpal tunnel syndrome however, other authors have not advocated the need for carpal tunnel decompression as a routine in any fracture type, age group or gender [2,27-30]. Whether to routinely perform carpal tunnel decompression in certain patients remains controversial and it remains our policy to select patients on a case-by-case basis.

16 patients developed features of CRPS at some point in their follow up. In order to avoid under reporting the incidence of CRPS, we included all patients who had any mention of CRPS type symptoms (e.g disproportionate or neurogenic type pain; trophic changes to the skin; persistent swelling) in their follow up records. There was no correlation between fracture type or period of immobilisation and the incidence of CRPS, however, the mean age of those with CRPS was younger (58.9 years) than the group as a whole. Also, 13 (81%) of these patients were female. This finding is in keeping with other reported incidences of CRPS after distal radius fracture [30]. We now use vitamin C prophylactically for CRPS prevention as recommended by Zollinger et al. [31]

It is not viable to statistically compare the DASH and MAYO scores of those with a complication and the general cohort, as 'complications' are an outcome in themselves, and these patients were not pre-defined for comparison. However, it can be inferred that those who sustain a complication are likely to have a worse functional outcome. Our overall complication rate is comparable or better than other reported treatment modalities including non-operative treatment [3,17], percutaneous fixation [16,31,32], volar locking plate fixation [5,18-21,33-38] and external fixation [15,18,33-35,38]. What is clear is that whilst this treatment modality offers good to excellent long-term results, with a low incidence of complication, when complications do occur, the functional impairment incurred is long lasting and hence surgery must be performed with great care and by well trained operators. On balance, we do not recommend routine removal of plates given that plate related complications in our series were few.

As this is a retrospective non-comparative study, we cannot disregard other treatment modalities for distal radius fracture. However, there is a growing body of evidence that supports volar locking plate fixation as the modality of choice when surgery is indicated. We feel that this study which is the largest of this kind supports this trend but do acknowledge that further high quality trials are necessary to better answer this contentious issue.

## Conclusions

This large series demonstrates good to excellent results in the majority of patients after volar locking plate fixation of the distal radius, with complication rates comparable to other non-operative and operative treatment modalities. Those who suffer a complication have a much worse functional outcome, hence, while we recommend this mode of fixation for distal radius fractures requiring operative intervention, utmost care must be exercised to prevent complications and identify them early.

## Competing interests

The authors declare that they have no competing interests.

## Authors' contributions

JP, AT and KG collected the data and compiled the patient database. They followed up the patients and wrote the paper. LB performed all statistical analysis. DSE & KJN were responsible for the conception of the study and supervision of the project. They proof read and amended the paper as necessary. All authors have read and approved the manuscript.
